# BMI, Overweight Status and Obesity Adjusted by Various Factors in All Age Groups in the Population of a City in Northeastern Brazil 

**DOI:** 10.3390/ijerph120404422

**Published:** 2015-04-22

**Authors:** Raquel Patrícia Ataíde Lima, Danielle de Carvalho Pereira, Rafaella Cristhine Pordeus Luna, Maria da Conceição Rodrigues Gonçalves, Roberto Teixeira de Lima, Malaquias Batista Filho, Rosália Gouveia Filizola, Ronei Marcos de Moraes, Luiza Sonia Rios Asciutti, Maria José de Carvalho Costa

**Affiliations:** 1 Graduate Program in Nutritional Sciences, Center for Health Sciences/NIESN—Interdisciplinary Studies in Health and Nutrition, Federal University of Paraíba, Castelo Branco, João Pessoa, PB 58059-900, Brazil; E-Mails: danicarvalhop@hotmail.com (D.C.P.); rafaellacristhine@yahoo.com.br (R.C.P.L.); 2 Postgraduate Program in Nutrition, Center for Health Sciences, Federal University of Paraiba, João Pessoa, PB, 58059-900, Brazil; E-Mails: raulceica@ig.com.br (M.C.R.G.); robtex@ibest.com.br (R.T.L.); rosaliafilizola@gmail.com (R.G.F.); mjc.costa@terra.com.br (M.J.C.C.); 3 Postgraduate Program in Mother and Child Health (IMIP), Instituto de Medicina Integral Prof. Fernando Figueira—IMIP, Board of Research, Boa Vista, Recife, PE 50070-550, Brazil; E-Mail: malaquias.imip@gmail.com; 4 Postgraduate Program in Decision Models and Health, Department of Statistics, Center of Exact and Natural Sciences, Federal University of Paraíba, João Pessoa, PB 58051-000, Brazil; E-Mail: ronei@de.ufpb.br; 5 Faculty of Medical Sciences, Department of Nutrition, João Pessoa, PB 58010-000, Brazil; E-Mail: luizaasciutt@terra.com.br

**Keywords:** obesity, schooling, income, lifestyle, total population

## Abstract

*Objective*: In Brazil, demographic, socioeconomic and epidemiological changes over time have led to a transition in nutritional standards, resulting in a gradual reduction of malnutrition and an increased prevalence of overweight and obese individuals, similar to the situation in developed countries in previous decades. This study assessed the body mass index (BMI) and the prevalence of an overweight status and obesity, adjusted for various factors, in a population in northeastern Brazil including all age groups. *Methods*: This is a cross-sectional population-based epidemiological study using single sampling procedure composed of levels. Given the heterogeneity of the variable “income” and the relationship between income, prevalence of diseases and nutrition, a stratified sampling on blocks in the first level was used. In this, city districts were classified by income into 10 strata, according to information obtained from IBGE. A systematic sampling was applied on randomly selected blocks in order to choose the residences that would be part of the sample (second level), including 1165 participants from all age groups. *Results and Discussion*: The prevalence of an overweight status or obesity was adjusted for demographic, socioeconomic and lifestyle variables. When the Chi-square test was applied, a relationship was observed between the prevalence of an overweight status or obesity and the age group, gender, educational level and income of the participants. Regarding lifestyle parameters, only smoking was associated with the prevalence of an overweight status or obesity, in both adults and in the total sample. The results for the following groups were significant (*p* < 0.05): the age group from 20 to 59 years, when the individual presented an educational level greater than or equal to high school; and the age group ≥ 60 years, when the individual was female. It is noteworthy that educational level and being female were significant in adjusting for the total population as major factors influencing an increased BMI, followed by the variables physical activity and family income. *Conclusions*: The adjusted results justify the adoption of intervention and prevention policies to combat these clinical conditions for the study population as a whole, particularly directed toward adults with higher education level as well as elderly females.

## 1. Introduction

Expansion of the prevalence of an overweight status and obesity is occurring in all age groups and all social strata [1], and 2.8 million people die worldwide every year due to these clinical conditions [2]. In Brazil, demographic, socioeconomic and epidemiological changes over time have led to a transition in nutritional standards, resulting in a gradual reduction of malnutrition and increased prevalence of an overweight status and obesity. This situation is similar to that in observed developed countries in previous decades [3].

Socioeconomic indicators such as income and educational level can influence the body weight of individuals. In developed countries, individuals with a low socioeconomic status are more likely to be obese compared with individuals with a high socioeconomic status [4]. In the United States, the difference in the obesity rates between the richest and poorest individuals is decreasing significantly [5], as corroborated by Popkin [6], who reported that the prevalence of obesity is also a serious problem in many low- and middle income countries.

The situation described above is in agreement with results of surveys carried out recently in Brazil on the influence of socioeconomic factors on the prevalence of an overweight status and obesity. With regard to education level, based on data from a study conducted at the national level (Surveillance of Risk Factors and Protection against Chronic Diseases by Telephone Interview—VIGITEL [7]), the influence of this factor on the frequency of an overweight status and obesity is significant among women as a function of their income. Additionally, according to the Family Budget Survey—POF [8], it was found that the prevalence of obesity was greater in adult women than in men, but no relationship was found with income, which showed a curvilinear distribution; *i.e.*, the highest prevalences were observed in middle income classes; however in the other age groups, the prevalence of this type of morbidity increased in higher income classes.

Therefore, in addition to the relationship between obesity and socioeconomic factors involving income and education [9], obesity is also related to factors such as age, sex and lifestyle (smoking, alcohol consumption and physical activity) [10,11,12]. According to Lee [13] childhood obesity is related to low physical activity level and a sedentary lifestyle.

There is no study in the literature associating BMI, an overweight status and obesity adjusted for socioeconomic, demographic and lifestyle factors in all age groups in the same population, allowing a deeper analysis of the influence of these factors on nutritional status. Thus, the present study aimed to evaluate the distribution of an overweight status, obesity and the BMI adjusted for socioeconomic, demographic and lifestyle factors in all age groups in the same population, which could contribute to better-targeted intervention strategies to combat these types of morbidity in populations with characteristics similar to those of the present study.

## 2. Participants and Methods

### 2.1. Study Design and Population

This study was part of a research project entitled “First diagnosis and intervention of food and nutritional situation and non-communicable diseases most prevalent in the population of João Pessoa, northeastern Brazil (I DISANDNT/JP)”, which was carried out from July 2008 to January 2010.

This cross-sectional population-based epidemiological study was conducted through stratified and systematic sampling, assessing the study population with the aid of Core R Development Team software [14], with 1165 individuals being selected for the study.

The total number of districts visited, corresponding to the five health districts of the city of João Pessoa, was 60, encompassing a total of 8338 blocks. Among these blocks, using Core R Development Team software [14], 274 blocks were randomly selected to be visited. Following area recognition, 253 visited blocks were computed, which comprised 722 randomly selected households. Regarding the estimate, 21 blocks were not visited after area recognition due to the presence of districts without defined households (e.g., lands, farms) and commercial districts. In each household visited, if the presence of a child, adolescent, adult or elderly individual was observed, they were invited to participate in the research. When there were two or more children, adolescents, adults or elderly residents in the household, only one individual per age group was randomly selected using dice.

**Figure 1:**
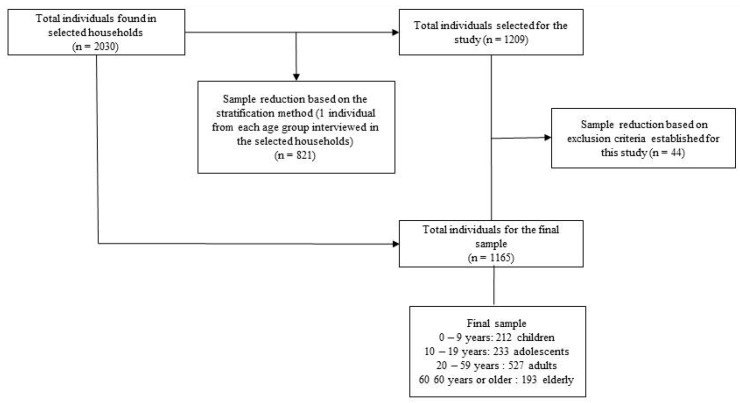
Flow diagram for sample definition.

When there were two or more individuals in each of these age groups living in the household, subjects were randomly chosen using instruments (data) for the selection of only one individual per group. If one or more individuals were not at home during the home visits, the team scheduled another visit in which these individuals would be at home. The inclusion criteria were as follows: individuals from all age groups, different socioeconomic levels, with or without chronic-degenerative diseases and either using drugs or not. The exclusion criteria were as follows: elderly individuals with neuropsychiatric disorders (n = 7), users of supplemental multivitamins, minerals, appetite suppressants and steroids (n = 29) and pregnant women (n = 8). This information is detailed in Figure 1.

Data were collected in home visits by a team that was properly trained after the completion of the pilot study. Questionnaires were employed to obtain socioeconomic, demographic, epidemiological and lifestyle information, and an anthropometric nutritional assessment was performed. More detailed information about the definition of the total sample and data collection are provided in a previously published study that was developed by part of the team conducting this research [15].

Individuals who agreed to participate in all stages of the research were asked to read and sign an “Informed Consent Form”. The project was approved by the Ethics Research Committee—Department of Health Sciences (CCS), Federal University of Paraíba, under protocol number 0493.

Family income was classified according to the median income as follows: less than the median or greater than or equal to the median. For purposes of data analysis, educational level was determined according to the Brazilian educational system, which in terms of school years is: 0 years (illiterate), up to 9 years (high school) and ≥9 years (high school to higher). Based on information about the schooling of the mother or the person responsible for feeding the family the relationship between this variable and nutritional status in the age group of children and adolescents aged 0 and 19 years wase established. As for the other age groups, the educational level of participants was used.

### 2.2. Body Measurements

Height and weight measurements were performed in triplicate, and the average of the three values was used. Children under two years were weighed using pediatric-type scales. To measure the weight of individuals over two years, a digital scale was used.

The length of children under two years of age was measured using a horizontal infantometer and a tape measure prepared by technicians from the Student Support Foundation (FAE), and the World Food Program was used for measuring the height of children over the age of 2 years.

The indicator used to assess the nutritional status of individuals from all age groups was the (BMI), according to the classification of the World Health Organization. For children and adolescents aged 0–9 and 10–19 years, the WHO classification published in 2006 [16] was employed, whereas for adults and seniors aged ≥20 years and ≥60 years, the WHO classification of 1995 [17] was used. The body mass index (BMI) was calculated as body weight (kilograms) divided by the square of body height (meters) [2] and was universally used to determine the prevalence of underweight, normal weight, overweight and obese statuses in all age groups. In this study, children, adolescents, adults and elderly people were evaluated by age group, using the following cutoff points for children and adolescents <3rd percentile = low BMI for age; ≥3rd percentile and <85th percentile = adequate BMI; ≥85th percentile and ≤97th percentile = overweight; and >97th percentile = obese; for the classification of adults and elderly people, the cutoff points were as follows: <18.5 kg/m^2^ = low BMI for age; n ≥ 18.5–<24.9 kg/m^2^ = adequate BMI; ≥25 and <29.9 kg/m^2^ = overweight; and ≥30 kg/m^2^ = obese.

The dietary survey was conducted with the aid of guidelines for home measures [18], employing the Quantitative Survey of Food Intake Frequency (QQFCA), which was validated using three 24-hour recalls applied at different time intervals for the female population of the city of João Pessoa/Paraíba/Brazil. This validation study was carried out in a partnership between the School of Public Health, University of São Paulo, and the Graduate Program in Nutritional Sciences, Federal University of Paraíba [19,20]. Individuals responded to a questionnaire regarding alcohol consumption, categorized as yes or no; in the case of children under 9 years, their guardians responded to the questionnaire; a similar procedure was adopted regarding smoking habits [21]. 

Individuals who performed regular physical activity at least 30 min per day and at least six times a week, or at least 150 min per week, were considered not sedentary [22]. For the age group between 0–9 years, their guardians responded regarding the performance of physical activity.

### 2.3. Statistical Analyses

Initially, the analysis of the characteristics of the sample population was expressed through descriptive statistics, represented by the simple frequency, using position measures such as the central tendency and dispersion (mean, standard deviation, range and percentage). Then, the data were checked for normality using the Lilliefors normality test, which is a derivation of the Kolmogorov-Smirnov test [23]. All statistical analyses were performed with the aid of Core R Development Team software [14]. For verify the existence of associations with variables, the Chi-square test was employed. A significance level of 5% was adopted to reject the null hypothesis.

To explain the BMI values of individuals related to the variables, multiple linear regression was applied for each age group and the total sample:

BMI = β0 + β1 school + β2 income + β3 sex + β4 physical activity
(1)

When adjustment was performed according to age groups, for adolescents, adults and elderly people, alcoholism and smoking were also considered.

BMI = β0 + β1 school + β2 income + β3 sex + β4 physical activity + β5 alcohol + β6 smoking (2)

For all statistical analyses, *p* values < 0.05 were considered significant.

## 3. Results

Data on the general, anthropometric, demographic, socioeconomic and lifestyle characteristics of the individuals in all age groups from I DISANDNT/JP are shown in Table 1.

**Table 1:** General characteristics of individuals from I DISANDNT/JP, for the municipality of northeastern Brazil (2008–2010).

		Mean	SD	Amplitude	N	%
**Demographic, socioeconomic and lifestyle characteristics**						
Sex	males	-	-	-	420	36
	females	-	-	-	745	64
Age (years)	0–9 years	4.52	2.94	9.00	212	18.1
	10–19 years	14.36	2.84	9.00	233	20.1
	20–59 years	38.64	11.41	39.00	527	45.3
	≥60 years	69.34	7.60	37.00	192	16.5
		32.63	22.84	97.00	1164	100.00
Schooling ^1^	Up to elementary school	-	-	-	553	47.5
						
						
	≥incomplete high school	-	-	-	612	52.5
					1165	100.00
Family income (R$) ^2^		1843.30	2159.54	18,000.00	1126	100.00
Smoking	yes	-	-	-	107	9.2
	no	-	-	-	1058	90.8
					1165	100.00
Alcohol consumption ^3^	yes	0.29	0.37	2.5	158	13.6
****	no	-	-	-	1006	86.4
****	****				1164	100.00
Practice of phys. Activity ^4^	yes	-	-	-	111	9.5
(5 times / week)	days/week	5.32	0.67	2.00	-	-
****	Duration activity/min	70.53	43.90	245	-	-
****	no	-	-	-	1054	90.5
****	****				1165	100.00
**Anthropometric characteristics**						
Weight(kg)		52.38	25.21	126	1095	-
Height(m)		1.40	0.42	1.85	1089	-
BMI (kg/m^2^)		23.57	6.31	49.83	1086	-
BMI classification ^*^						
	Low weight/normal weight	19.41	3.24	13.24	572	52.7
	Overweight	27.16	1.43	5.03	305	28
	Obesity	33.85	3.56	21.30	209	19.2

Notes: ^1^ <elementary school, corresponding to 9 years or less of schooling, and >incomplete high school, corresponding to more than 9 years of schooling; ^2^ Median family income, R$1000.00, or $492.02; ^3^ Alcohol consumption: only for aged adults and elderly; ^4^ five times per week, at least 30 min. * According to the WHO (1995) and WHO (2007). Abbreviations: SD—standard deviation, BMI—body mass index, I DISANDNT/JP—“First diagnosis and intervention of food and nutritional situation and non-communicable diseases most prevalent in the population of João Pessoa, northeastern Brazil”.

The total sample (n = 1165) consisted predominantly of women. The majority of the population was eutrophic, and the prevalence of an overweight status or obesity was 47.3%, more than half of which consisted of overweight individuals.

The prevalence of an overweight status and obesity in the sample distributed according to sex, age group and socioeconomic status (including income and educational level) is presented for the total sample and distributed by age group in Table 2 as well as in relation to lifestyle parameters including smoking, physical activity and alcohol consumption in the total sample.

In the total sample, the prevalence of an overweight status and obesity was related to sex, educational level, income, and smoking habits. Additionally, when the sample was divided by age group, a relationship with sex, educational level (among those who completed elementary school) and family income was found.

For the total sample, a relationship of the prevalence of an overweight status and obesity with sex, educational level, income and smoking habits was observed. When the sample was distributed by age groups, a relationship between sex, educational levels among those who had completed high school and family income was detected.

Regarding the relationship between the prevalence of an overweight status or obesity and lifestyle variables, the only detected association was with smoking habits in the total sample, and alcohol consumption was higher in the elderly (*p* value > 0.02). 

**Table 2:** Relationship between the nutritional status of individuals and socioeconomic, demographic and lifestyle indicators for all of individuals from I DISANDNT/JP, for the municipality of northeastern Brazil (2008–2010).

Variables		Low weight/normal weight		Overweight		Obesity		Total	*p*-Value
		n	%	n	%	n	%	N	
Age group	0–9	139	68.47	40	19.7	24	11.82	203	0.0000 *
	10–19	158	72.81	42	19.35	17	7.83	217	
	20–59	211	43.78	160	43.19	111	23.03	482	
	≥60 years	64	34.78	63	34.24	57	30.98	184	
**Sex**	Males	224	60.4	97	26.14	50	13.5	371	0.0000 *
	Females	351	48.89	208	28.97	159	22.14	718	
Sex: male (According to age group)	0–9	75	68.18	21	19.09	14	12.73	110	0.0000 *
	10–19	72	72.00	22	22	06	6.00	100	
	20–59	47	48.96	32	33.33	17	17.71	96	
	≥60 years	25	41.67	22	36.67	13	21.66	60	
Sex: female (According to age group)	0–9	64	68.82	19	20.43	10	10.75	93	0.0000 *
	10–19	85	73.28	20	17.24	11	9.48	116	
	20–59	164	42.49	128	33.16	94	24.35	386	
	≥60 years	38	30.90	41	33.33	44	35.77	123	
**Schooling ^1^**	Up to elementary school	281	53.12	133	25.14	115	21.74	529	* 0.0025
	≥incomplete high school	290	52.16	172	30.93	94	16.91	556	0.0000 *
Schooling according to age group									
Up to elementary school	0–9	84	74.34	16	14.16	13	11.50	113	0.0000 *
	10–19	80	74.07	19	17.59	9	8.33	108	
	20–59	81	42.86	54	28.57	54	28.57	189	
	≥60 years	36	30.25	44	36.98	39	32.77	119	
≥incomplete high school	0–9	55	61.11	24	26.67	11	12.22	90	0.5515
	10–19	78	71.56	23	21.10	8	7.34	109	
	20–59	130	44.37	106	36.18	57	19.45	293	
	≥60 years	27	42.19	19	29.69	18	28.12	64	
**Family income (R$)^2^**	≤1000	304	54.19	110	19.61	147	26.20	561	0.0000 *
	>1000	252	51.01	153	30.97	89	18.02	494	
Family income according to age group									
<Median	0–9	86	70.5	21	17.2	15	12.3	122	0.0000 *
	10–19	89	72.35	26	21.13	8	6.50	123	
	20–59	103	33.1	70	29.3	66	27.6	239	
	≥60 years	24	32.87	28	38.5	21	28.76	73	
≥Median	0–9	47	46.4	18	26.08	5	7.24	69	>0.0000 *
	10–19	63	71.6	16	18.18	9	10.22	88	
	20–59	105	45.06	87	37.33	41	17.60	233	
	≥60 years	36	35.3	32	31.37	34	33.33	102	
Smoking	no	523	52.88	281	28.41	185	18.71	989	0.0471 *
	yes	49	50.52	24	24.74	24	24.71	97	
Alcohol consumption	no	498	53.72	257	27.72	172	18.56	927	0.1785
	yes	73	46.20	48	30.38	37	23.42	158	
Pract. physical activity	no	526	53.24	273	27.63	189	19.13	988	0.4611
(5 times/week)	yes	46	46.94	32	32.65	20	20.41	98	

Notes: * *p* < 0.05; ^1^ < elementary school, corresponding to 9 years or less of schooling, and ≥incomplete high school, corresponding to more than 9 years of education, ^2^ Median family income, R$1000.00, or $492.02. Abbreviations: SD—standard deviation, BMI—body mass index.

Table 3 shows the results of the multiple regression test based on models proposed to adjust the variables employed in this study by age group and in the total population. 

**Table 3:** Multiple regression analysis between socioeconomic, demographic and lifestyle indicators and the BMI of individuals of all ages.

Multiple Regression				
**Adjustment 1: all age groups**				
	**Coefficient**	**CI** **95%**	**Statistics** ***t***	***p*-Value**
Intercept	16.41	16.41 ± 0.71	22.95	>0.0000 *
Schooling	3.21	3.21 ± 0.36	8.83	>0.0000 *
Income	0.25 × 10^−3^	0.25 × 10^−3^ ± 0.09× 10^−3^	2.95	0.0032 *
Sex	2.96	2.96 ± 0.38	7.69	>0.0000 *
Physical activity	0.92	0.92 ± 0.43	2.12	0.0344 *
**Adjustment 2: 0 to 9 years**				
	**Coefficient**	**CI** **95%**	**Statistics** ***t***	***p*-Value**
Intercept	17.4	17.4 ± 0.7	24.65	>0.0000 *
Schooling	−0.09	−0.09 ± 0.44	−0.20	0.8410
Income	0.03 × 10^−3^	0.03 × 10^−3^ ± 0.13 × 10^−3^	0.24	0.8140
Sex	−0.58	−0.58 ± 0.41	−1.39	0.1660
Physical activity	0.03	0.03 ± 0.71	0.04	0.9650
**Adjustment 3: 10 to 19 years**				
	**Coefficient**	**CI** **95%**	**Statistics** ***t***	***p*-Value**
Intercept	18.33	18.33 ± 1.18	15.52	>0.0000 *
Schooling	0.9	0.9 ± 0.64	1.39	0.1650
Income	0.17 × 10^−3^	0.17 × 10^−3^ ± 0.15 × 10^−3^	1.13	0.2590
Sex	0.91	0.91 ± 0.65	1.39	0.1670
Physical activity	1.11	1.11 ± 0.71	1.57	0.1118
**Adjustment 4: 20 to 59 years**				
	**Coefficient**	**CI** **95%**	**Statistics** ***t***	***p*-Value**
Intercept	22.91	22.91 ± 1.30	17.56	>0.0000 *
Schooling	2.48	2.48 ± 0.51	4.89	>0.0000 *
Income	0.00	0.00 ± 0.12 × 10^−3^	0.01	0.9946
Sex	1.10	1.10 ± 0.64	1.72	0.0866
Physical activity	−0.42	−0.42 ± 0.61	−0.70	0.4857
Smoking	−0.29	0.29 ± 0.7	−0.43	0.6662
Alcohol consumption	1.02	1.02 ± 1.89	0.54	0.5897
**Adjustment 5: 60 years or older**				
	**Coefficient**	**CI** **95%**	**Statistics** ***t***	***p*-Value**
Intercept	23.81	23.81 ± 1.61	14.75	>0.0000 *
Schooling	1.51	1.51 ± 0.78	1.93	0.0552
Income	0.13 × 10^−3^	0.13 × 10^−3^ ± 0.16 × 10^−3^	0.00	0.3962
Sex	1.72	1.72 ± 0.81	2.11	0.0362 *
Physical activity	−0.35	−0.35 ± 0.80	−0.45	0.6569
Smoking	−1.35	−1.35 ± 1.26	−1.07	0.2880
Alcohol consumption	−0.91	−0.91 ± 1.29	−0.71	0.4807

Note: * *p* < 0.05.

In the final models, *i.e.*, the last statistical model applied to these variables, *p* values < 0.05 were obtained for the following groups: for the age group from 20–59 years, when an individual exhibited an educational level greater than or equal to high school, the average BMI was increased by 2.48 kg/m^2^; and for the age group ≥ 60 years, when the individual was female, the BMI was increased by 1.72 kg/m^2^ on average. With regard to all age groups, when the educational level was greater than or equal to high school, the average BMI was increased by 3.21 kg/m^2^. For children aged 0–9 years and adolescents aged 10–19 years, the educational level used in the analysis including all age groups was that of parents or guardians. When the individual was female, the BMI was increased by 2.96 kg/m^2^. When family income increased by 1 real (the Brazilian currency), the average BMI was increased by 0.25 kg/m^2^, and when the individual performed physical exercise, the average BMI was increased by 0.92 kg/m^2^.

## 4. Discussion

In this study, by applying the chi-squared test, a positive relationship was found between the prevalence of an overweight status or obesity, based on the BMI, and the following types of variables: demographic, when the sample was distributed by age and sex; socioeconomic, with regard to educational level, when the sample was distributed by schooling years; family income, when the sample was distributed based on the median income by age group and in the total sample; and lifestyle, in regard to smoking habits in the total sample.

Following multivariate adjustment, these relationships remained for the total sample, but not when the sample was distributed by age group, with two exceptions: first, regarding educational levels and the relationship found between the age group from 20–59 years and increased BMI, it was shown that an increasing educational level was associated with an increase of the BMI by 2.48 kg/m^2^; and second, for females aged ≥ 60 years, or when an individual belonged to this sex and this age group, the BMI was increased by 1.72 kg/m^2^. After adjustment, no influence of smoking habits and alcohol consumption was observed (Table 3). It has been reported that a low educational level is a risk factor for obesity, but in a study carried out by Sabanayagam *et al.* [24], this relationship was observed only for females; in males, higher educational levels were associated with a higher prevalence of obesity. According to Dinsa *et al.* [25], in low-income countries, obesity is more prevalent among groups with a higher socioeconomic status (*i.e.*, those with a higher income or educational level), thus corroborating the findings of the present study regarding educational level, but not income. One explanation for this result may be that more energy-dense food products tend to have a lower cost in developing countries [25].

There are currently no available studies in the literature associating BMI and the prevalence of an overweight status or obesity adjusted for demographic, socioeconomic and lifestyle factors in the same population, in both the total population and in the sample distributed by all age groups.

Studies on the prevalence of an overweight status or obesity and measurements of anthropometric data distributed by age group are also scarce and have produced conflicting results. Hence, it is pertinent to mention that for comparative purposes, the factors that interfere with this prevalence, although similar, are different among populations.

Only one study was found in literature for the age group from 2–19 years [25], which is close to the first group examined in the present study, from 0–20 years, and the prevalence of obesity in this age group was 19.65% higher than in developing countries (1%–28%) [25] and in the United States 16.9% [26], most likely due to different demographic, socioeconomic and lifestyle factors. Furthermore, in the last population-based study carried out in Brazil, a prevalence of 19.2% was observed, while the prevalence was 14.5% in the northeastern region in the age group from 5 to 19 years—POF 2008–2009 [8]. Biro and Wein [27] explain the increasing prevalence of an overweight status and obesity in this age group based on complex interactions between genetic and environmental factors.

In the United States, the prevalence of obesity in adults aged 20–59 years was reported to be 35.7% [26]. In Brazil, according to data from the Surveillance of Risk Factors and Protective against Chronic Diseases by Telephone Survey—VIGITEL [7], in the age group equal to or greater than 18 years, the overall prevalence of obesity was 15%. According to POF (2008–2009) [8], in individuals aged 20 years or more, this value was 14.8%, and in the city of João Pessoa, where the present study was carried out, it was 23.02% for this age group, similar to the value obtained in the study by Ogden *et al.* [26], while it was 16.3%, according to VIGITEL [7].

Studies on the prevalence of obesity in the elderly are even scarcer. In the United States, this prevalence was reported to be 39.7% [25], which considered to be the highest value compared with age groups beginning at 2 years. In this study, the prevalence of an overweight status or obesity in the elderly was 36.92% or 30.76%, respectively. According to Marinos [28], this increase in BMI with age is due to the reduction of the total daily energy expenditure, among other factors.

When distributed by sex, the prevalence of obesity in adults ranges from 3% to 30% for men and from 1% to 50% for women in developing countries [24], in agreement with results shown in Table 2 for the total sample, and females exhibit the highest prevalence of obesity, at 22.1%, except in the age group from 0–9 years, in which the highest prevalence occurs among males. With regard to age, by which most of the variables that were components of the survey were distributed, the prevalence of obesity was lower in the age group from 10 to 19 years, most likely due to the increased height and decreased weight of adolescents [29], except with respect to the sample distribution by household income greater than the median. For an overweight status, the prevalence was higher than obesity in more than 80% of the prevalence found in the different variables.

With regard to the relationships detected between nutritional status and different variables, relationships were observed between nutritional status and the following parameters: age group, indicating that the higher the age group, the higher the prevalence of an overweight status and obesity, most likely due to significant changes in body composition and metabolism [30,31]; sex, indicating that females show the highest prevalence of an overweight status and obesity in the different age groups, possibly due to the interference of factors such as an increased fat mass in puberty [27]; responsibility for decisions about the quantity and quality of food provided to the family made by adults, which and can lead to food insecurity [32]; and greater accumulation of visceral fat in the elderly, with an increased life expectancy [17].

Regarding the relationship between nutritional status and socioeconomic variables in the total sample and among individuals presenting an educational level up to high school, the lower the educational level, the higher the prevalence obesity or an overweight status was observed to be. These results corroborate data obtained by Yoo *et al.* [33], who reported a higher prevalence of obesity in individuals with lower educational levels. Additionally, these findings are in accord with those of Zhang and Wang [34] to some extent, who observed that educational level is a better predictor of obesity than income in research conducted in the United States in men and women aged 25 to 64 years.

These considerations are important because obesity is considered to be a problem only in developed countries. However, its prevalence in developing countries is increasing. According to the WHO [2], approximately 35 million overweight children live in developing countries, compared with only 8 million in developed countries. In a study conducted in the United States, it was found that the highest obesity rates occurred among lower income groups [35], corroborating the results of Jones-Smith *et al.* [36], who reported that a low socioeconomic status was associated with a higher prevalence of obesity, as well as the results of the present study. However, with regard to the relationship between the prevalence of an overweight status and family income, an opposite situation was observed, with a greater prevalence of an overweight status being found in higher income groups, as reported by Neuman *et al.* [37]. Additionally, according to Monteiro *et al.* [3], in developing countries, the prevalence of obesity is greater in higher income populations, as observed in the present study, only adjusting variables in relation to an increasing BMI. 

However, caution is recommended in this type of discussion because there is no consensus regarding the relationship between income and nutritional status, as this relationship will depend on the characteristics of the study population. For example, in national surveys such as the Household Budget Survey (POF 2008–2009) [8], in children and adolescents, the prevalence of an overweight status and obesity shows a strong increase in association with income. In contrast, in adults, this increase is observed only in males, whereas in females, the highest prevalence occurs in the middle income class.

The values related to lifestyle variables such as physical activity, alcohol consumption and smoking habits were quite low when the sample was distributed by age group. These relationships were therefore studied only in the total sample, representing a limitation of this study. Although there was no relationship observed between physical activity and nutritional status, the increased prevalence of an overweight status and obesity remained even among those who practiced physical activity, justifying the need for dietary interventions associated with physical activity to achieve beneficial weight reduction results for overweight and obese adults [38] as well as for the general population. When referring children, Lee [13] also found no significant relationship in children between a sedentary lifestyle and anthropometric indicator. Lloyd and Wyatt [39] suggest programs involving schools and children to have improvements in children’s weights. Another factor implicated in the obesity epidemic, especially in children and adolescents is the availability of cheap foods, such as fast food [40], a factor that was not consider in this study become**s** a limitation.

Corroborating the results obtained by Baek and So [41] in research on adolescents, in which there was no significant difference found, and the results of studies on adults and elderly people [41], the present study did not reveal any relationship between alcohol consumption and nutritional status. However, alcohol consumption appears to alter nutritional status [42]. In British adults, it was found that an increased BMI resulted from alcohol consumption and sedentary behavior [42], which suggests that obesity is associated with lifestyle [42] However, although alcohol is a source of energy with a high-energy content, providing 7.1 kcal/g, it is still controversial whether alcohol consumption is a risk factor for an overweight status and obesity [42]. For this reason, further studies are needed to better understand the interaction between alcohol consumption and obesity [42].

Smoking was the only variable associated with lifestyle that was found to be related to nutritional status, with a greater prevalence of obesity being observed among smokers, but a lower prevalence of an overweight status. This finding corroborates results obtained by Wadden and Stunkard [43], who reported that smoking increases obesity, whereas this relationship was not observed when the BMI value was adjusted.

The strengths of this study include its stratified and systematic sampling design, allowing representation the population of the studied city, and the fact that it employed recent data. It is noteworthy that the use of a validated manual for the study population elaborated through actual representation of measures of food in the home facilitated a more accurate quantification of the size of alcohol servings ingested. Another positive aspect of this study was the home visits and the weekly meetings carried out with the entire team, with project coordination throughout the training period and during data collection, encouraging standardization of the methodology used throughout the research. In addition to the weight and height of individuals, using the BMI to assess nutritional status is justified because despite the limitations of this index, Dinsa *et al.* [25] found no differences when using BMI when compared with the waist circumference and waist-hip ratio. And as another limit of the study has included the absence of the relationship between ethnicity/race and socioeconomic level since, according to Shavers [44], socioeconomic status is often touted as a contributor to the disparity in health observed among racial/ethnic minorities.

## 5. Conclusions

These results contribute to clarify the relationships between the prevalence of an overweight status or obesity and BMI adjusted for socioeconomic, demographic and lifestyle variables in the population. The adjusted results justify the adoption of intervention and prevention policies to combat these clinical conditions for the study population as a whole, particularly directed toward adults with higher education level as well as elderly females.
